# Nuclear factor erythroid 2-related factor 2 promotes radioresistance by regulating glutamate-cysteine ligase modifier subunit and its unique immunoinvasive pattern

**DOI:** 10.17305/bb.2024.10184

**Published:** 2024-06-01

**Authors:** Zhaoyuan Xue, Yiliyaer Nuerrula, Yilidana Sitiwaerdi, Mayinur Eli

**Affiliations:** 1 Department of Oncology, The First Affiliated Hospital of Xinjiang Medical University, Urumqi, China

**Keywords:** Nuclear factor erythroid 2-related factor 2 (NRF2), glutamate-cysteine ligase modifier subunit (GCLM), radioresistance, ferroptosis, macrophage M2

## Abstract

The enzyme glutamate-cysteine ligase modifier subunit (GCLM) serves as the initial rate-limiting factor in glutathione (GSH) synthesis. GSH is the preferred substrate for glutathione peroxidase 4 (GPX4), directly impacting its activity and stability. This study aims to elucidate the expression of GCLM and its correlation with the nuclear factor erythroid 2-related factor 2 (NFE2L2), commonly referred to as NRF2, in esophageal squamous cell carcinoma (ESCC) and further investigate the potential signaling axis of radiotherapy resistance caused by NRF2-mediated regulation of ferroptosis in ESCC. The expression of NRF2, GCLM, and GPX4 in ESCC was analyzed by bioinformatics, and their relationship with ferroptosis was verified through cell function experiments. Their role in radioresistance was then investigated through multiple validation steps. Bioinformatics analysis was employed to determine the immune infiltration pattern of NRF2 in ESCC. Furthermore, the effect of NRF2-mediated massive macrophage M2 infiltration on radiotherapy and ferroptosis was validated through in vivo experiments. In vitro assays demonstrated that overactivated NRF2 promotes radioresistance by directly binding to the promoter region of GCLM. The Tumor Immune Estimation Resource (TIMER) and quanTIseq analyses revealed NRF2 enrichment in M2 macrophages with a positive correlation. Co-culturing KYSE450 cells with M2 macrophages demonstrated that a significant infiltration of macrophages M2 can render ESCC cells resistant to radiotherapy but restore their sensitivity to ferroptosis in the process. Our study elucidates a link between the NRF2-GCLM-GSH-GPX4 signaling axis in ESCC, highlighting its potential as a therapeutic target for antagonistic biomarkers of resistance in the future. Additionally, it provides a novel treatment avenue for ESCC metastasis and radioresistance.

## Introduction

Esophageal squamous cell carcinoma (ESCC) is a highly heterogeneous disease, with patients exhibiting diverse biological, clinical, genetic, and environmental characteristics [1, 2]. It ranks as the eighth most common cancer worldwide and the sixth leading cause of cancer-related deaths. Recent years have seen a rapid increase in ESCC incidence, affecting over 500,000 individuals annually. Due to the atypical early symptoms, the majority of patients receive diagnoses at intermediate or advanced stages, obstructing surgical intervention. Treatment for locally advanced ESCC typically encompasses a variety of comprehensive treatment methods, including chemoradiotherapy and surgery, while palliative care, including radiotherapy, is reserved for progressive stages such as metastatic or recurrent ESCC. Radiotherapy has been recognized as one of the standard treatment options for clinical ESCC. Nonetheless, resistance to radiotherapy often develops, leading to tumor recurrence and metastasis, thereby compromising treatment outcomes [3, 4]. Consequently, there is an urgent need for accurate biomarkers to predict and enhance the sensitivity of esophageal cancer to radiotherapy.

As a modification subunit, the glutamate-cysteine ligase modifier subunit (GCLM) effectively controls cellular glutathione (GSH) levels by regulating its affinity with glutamate through the heavy-chain glutamate-cysteine ligase (GCLC). Literature reports reveal that GCLM plays a pivotal role in drug resistance across a variety of cancers, including lung [5], breast [6], liver [7], ovarian [8], and bladder [9] cancers, where its upregulated expression is mostly linked to drug resistance. Additionally, the overexpression of GCLM has been identified to inhibit ferroptosis, exhibiting a significant correlation with patient treatment responses [10, 11].

In a preceding study, we conducted whole-genome exome sequencing on 120 patients with ESCC and identified 13 tumor-specific genes with high-frequency mutations [12]. Among these, the nuclear factor erythroid 2-related factor 2 (*NFE2L2*), commonly referred to as *NRF2*, is involved in oxidative stress, allowing cancer cells to develop high resistance to reactive oxygen species (ROS) and leading to cancer cell metabolic reprogramming. Subsequently, our research group examined the differential expression of the *NRF2* gene in cancerous and adjacent non-cancerous tissues from 174 ESCC patients at our institution using immunohistochemistry [13]. Subsequent functional analyses indicated that NRF2 overexpression correlates with the proliferation, invasion, and migration of esophageal cancer cells, acting as a tumor-promoting factor. Under normal circumstances, NRF2 activity is regulated by the kelch-like ECH-associated protein 1 (Keap1) in a stress-dependent manner, serving as a key regulator of redox homeostasis. However, extensive evidence suggests that the aberrant activation of the Keap1-NRF2 pathway in tumor cells leads to persistently elevated NRF2 levels, contributing to chemoradiotherapy resistance across various cancers [14, 15]. Notably, the overactivation of NRF2 in cancer cells has been linked to the inhibition of ferroptosis [16]. Nonetheless, the potential connection between radiotherapy resistance mediated by NRF2 through ferroptosis and its relation to GCLM in ESCC remains to be elucidated.

Tumor-associated macrophages (TAMs), defined as either the classical phenotype (known as M1) or the surrogate phenotype (known as M2), represent a double-edged sword in the tumor immune microenvironment (TIME) [17]. It has been established that macrophages can initiate multiple signaling pathways involved in ferroptosis, while ferroptosis products, in turn, can regulate TAM polarization [18, 19]. These interactions suggest that macrophage-ferroptosis crosstalk could significantly influence tumor cell development, highlighting the importance of a thorough understanding of this interaction.

Although NRF2’s role in regulating key signaling processes of ferroptosis is recognized, and extensive research links NRF2 with the proliferation, invasion, and migration of ESCC as an oncogenic factor, the specific mechanism of NRF2 in modulating ferroptosis to confer radiotherapy resistance in ESCC remains unexplored. Moreover, there is a lack of in vitro studies investigating the potential of biological behaviors such as targeting NRF2 to enhance ferroptosis in esophageal cancer cells and improve radiotherapy outcomes. We are determined to explore new molecular targets within ferroptosis to increase radiosensitivity by targeting ferroptosis modulators, thereby providing a theoretical foundation for improved, individualized treatment strategies for esophageal cancer.

## Materials and methods

### Cell lines and ferroptosis drugs

The human ESCC cell lines KYSE150 and KYSE450, all obtained from the First Affiliated Hospital of Xinjiang Medical University, underwent cellular short tandem repeat (STR) identification before utilization. These cells were cultured in Dulbecco’s Modified Eagle Medium (DMEM) (Gibco, C11995500BT, USA) with 10% fetal bovine serum (Sigma, F7524, USA) and 1% penicillin–streptomycin antibiotics (HyClone, SV30010, USA) within a 37 ^∘^C, 5% carbon dioxide incubator (Thermo Fisher Scientific, Massachusetts, USA). To verify that ferroptosis can occur after radiotherapy, we used the ferroptosis inhibitor ferrostatin-1 (Fer-1) (HY-100579, MedChemExpress, USA). For NRF2 overexpression, the NRF2 agonist tert-butylhydroquinone (TBHQ) (HY-100489, MedChemExpress, USA), which is a widely used NRF2 activator, was used. To verify whether the KYSE450 cell lines, which acquire a tendency to metastasis, exhibit heightened sensitivity to ferroptosis inducers, erastin (HY-15763, MedChemExpress, USA) was utilized. To strengthen our conclusions, we used an NRF2 inhibitor from another group, brusatol (HY-19543, MedChemExpress, USA).

### Western blot assay and antibodies

The cells were lysed using a lysis buffer (Beyotime Biotechnology, Nanjing, China) supplemented with protease inhibitor cocktails. The nuclear protein extraction kit (Beyotime Biotechnology) and the bicinchoninic acid (BCA) protein detection kit (Thermo Fisher Scientific) were employed for protein extraction and concentration determination, respectively. The proteins were identified using antibodies against NRF2 (1:1000 dilution, 80593-1-RR, Proteintech), GCLM (1:1000 dilution, R382192, ZEN-BIOSCIENCE), glutathione peroxidase 4 (GPX4) (1:1000 dilution, ab125066, Abcam), snail family transcriptional repressor 1 (Snail) (1:1000 dilution, 3879T, Cell Signaling Technology), epithelial cadherin (E-cadherin) (1:1000 dilution, 3195T, Cell Signaling Technology), acyl-CoA synthetase long-chain family member 4 (ACSL4) (1:1000 dilution, R24265-5, ZEN-BIOSCIENCE), and β-actin (1:5000 dilution, bs-0061R, Bioss). The molecular weight of proteins was determined using a prestained protein marker (26,616, Thermo Fisher Scientific, USA). Quantitative analysis of protein bands was performed using ImageJ software, with the gray value ratio of the target protein to the reference protein serving as the metric for analysis.

### Quantitative polymerase chain reaction (qPCR)

Total RNA was extracted from ESCC cell lines using the RNAsimple Total RNA kit (Foregene, Cheng Du, China). A quantity of 500 ng of total RNA was reverse transcribed using the complementary DNA (cDNA) Reverse Transcription Kit (Evo-M-MLV, Accurate, China) in a 10 µL reaction volume. Following amplification, the resultant cDNA underwent qPCR with SYBR Green, ensuring results were considered reliable only when a single, distinct peak occurred at the same temperature as that of the corresponding primer. The CT values of reliable expression results were analyzed, calculated, and recorded. The expression levels of relevant genes were determined using the 2^-ΔΔCT^ method. The included qPCR primers are presented in Table 1.

**Table 1:** The included qPCR primer sequences

**Gene**	**Primer sequences**
*CCL22*	Forward: 5-GAGCATGGATCGCCTACAG-3 Reverse: 5-CAGACGGTAACGGACGTAATC-3
*CD206*	Forward: 5-GCAAAGTGGATTACGTGTCTTG-3 Reverse: 5-CTGTTATGTCGCTGGCAAATG-3
*GAPDH*	Forward: 5-CAACGGATTTGGTCGTATTGG-3 Reverse: 5-TGACGGTGCCATGGAATT T-3

qPCR: Quantitative polymerase chain reaction; CCL22: Chemokine (C-C motif) ligand 22; CD: Cluster of differentiation; GAPDH: Glyceraldehyde 3-phosphate dehydrogenase.

### Indicators of ferroptosis detection

The KYSE150 and KYSE450 esophageal cancer cell lines were pretreated with Fer-1 and TBHQ, or left untreated for 24 h, respectively. Ten hours post-radiotherapy, cells underwent digestion, centrifugation, and collection. Subsequently, the content of ROS, malondialdehyde (MDA), and GSH (all from Solarbio, Beijing, China) was assessed, following the instructions provided by the respective kits.

### Transmission electron microscopy

A small sample was collected using a cell scraper and fixed in 4% paraformaldehyde, followed by washing in buffer. The sample was then fixed in 1% buffered osmium and stained with 1% filtered uranyl acetate. After undergoing dehydration and embedding, the samples were incubated in a 60 ^∘^C oven for approximately three days. Ultrathin sections were prepared and examined under a microscope.

### Cell proliferation assay

Cell Counting Kit-8 (CCK8) assays (Bioss, Beijing, China) were conducted to determine cell viability. The cells were seeded at a density of 5,000 cells/well in a 96-well plate and cultured at 37 ^∘^C in a 5% carbon dioxide atmosphere. The KYSE150 and KYSE450 cells underwent radiotherapy using a linear accelerator (Varian, California, USA) at a dose rate of 400 cGy/min. Cell viability was determined according to the instructions, and the optical density (OD) value was measured using a microplate reader (Thermo Fisher Scientific, Massachusetts, USA). Cells were then pretreated with 5 µM Fer-1 and 5 µM TBHQ, or received no treatment, for 24 h prior to radiotherapy. This procedure was repeated based on the optimal radiation dose identified.

### Co-culture

THP-1 cells were seeded in the upper chamber of a transwell 6-well plate and treated with 100 ng/mL phorbol 12-myristate 13-acetate (PMA) for 48 h to induce differentiation into adherent M0-type macrophages. These M0-type macrophages were then incubated with 20 ng/mL interleukin (IL)-4 and 20 ng/mL IL-13 to further differentiate into M2-type macrophages. On the day before polarization completion, KYSE450 cells were seeded in the lower chamber to allow for overnight adhesion. Following polarization, each transwell upper chamber was transferred to a 6-well plate containing KYSE450 cells. The polarized macrophages and esophageal cancer cells were then cultured in a serum-free medium for 24 h.

### Cell scratch assay

According to the method provided above, both the co-culture and the KYSE450 cell line without any treatment were seeded in the same 6-well plate. After allowing the cells to adhere, they were subjected to radiotherapy at a dose of 4 Gy. Following irradiation, the adherent cells were vertically scratched on the bottom of the 6-well plate using a pipette tip. Subsequently, the old medium was discarded, and the cells were washed with phosphate-buffered saline (PBS). Three random fields of view were then photographed under an inverted microscope, marking the starting point (0 h) of the scratch. Next, serum-free DMEM medium was added to each well, and the cells were observed under an inverted microscope for 24 h to track cell migration in the same field of view as at the start of the scratch (0 h). Photographs were taken, and the mean distance between cells was calculated to compare the rate of scratch healing among the cells.

### Bioinformatics

The RNA-sequencing expression profiles (level 3) and corresponding clinical information for 1538 samples were obtained from The Cancer Genome Atlas (TCGA) dataset (https://portal.gdc.com). Ferroptosis-related genes were extracted from Liu et al.’s study, “Systematic analysis of the aberrances and functional implications of ferroptosis in cancer” [20]. Initially, the 3D structures of NRF2 and GCLM proteins were retrieved from the Protein Data Bank (PDB) database. The spatial interaction between them was examined using computational methods, predicting their binding mode and affinity. Docking results were generated using Z-domain docking (ZDOCK) and scored accordingly. To validate the reliability of immune score evaluation, the immuneeconv package was utilized. Analysis and visualization were conducted using the R software ggClusterNet package. Potential response to immune checkpoint blockade (ICB) was predicted using the Tumor Immune Dysfunction and Exclusion (TIDE) algorithm. All the aforementioned analyses and R packages were performed using R software version v4.1.3 (R Foundation for Statistical Computing, 2022). A *P* value < 0.05 was considered statistically significant.

### Statistical analysis

Results conforming to the normal distribution of metric data are presented as mean ± standard deviation, while results not conforming to the normal distribution are presented as medians. The Wilcoxon test is employed for two independent samples that do not follow a normal distribution or have uneven variance. For multi-sample data with uniform variance and normal distribution, Analysis of Variance (ANOVA) is selected. In cases where the variance is uneven or does not follow a normal distribution, the Kruskal–Wallis test is used. GraphPad Prism 9 software was utilized for data analysis and graphical representation. A *P* value < 0.05 was considered statistically significant.

**Figure 1.**
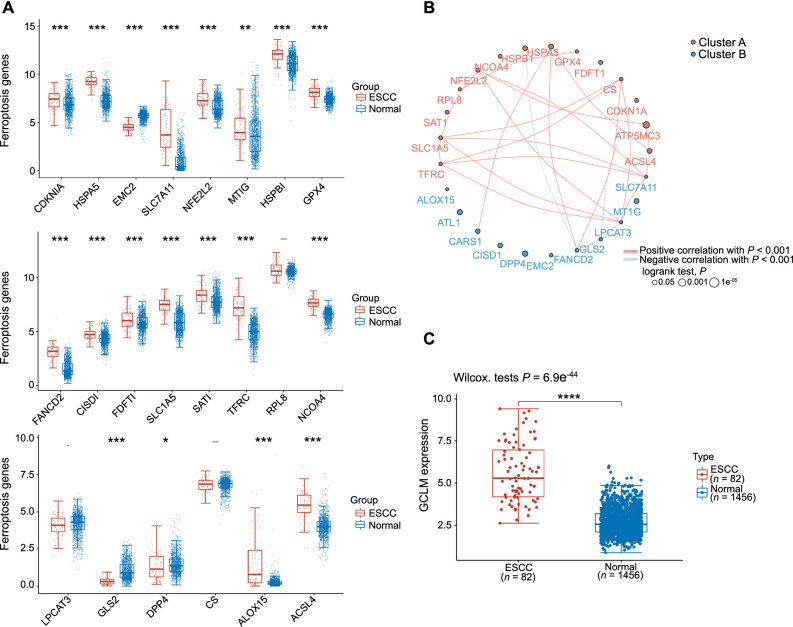
**Ferroptosis-related gene expression and protein interaction network in ESCC.** (A) Graphical representation of ferroptosis-related gene expression levels in ESCC vs normal samples; (B) Correlation circle plot depicting the relationship between ferroptosis-related genes in ESCC; (C) Box plot showcasing the expression levels of GCLM in ESCC in comparison to normal tissues. *P < 0.05; **P < 0.01; ***P < 0.001; ****P < 0.0001. ESCC: Esophageal squamous cell carcinoma; GCLM: Glutamate-cysteine ligase modifier subunit; CDKN1A: Cyclin dependent kinase inhibitor 1A; HSPA5: Heat shock protein family A member 5; EMC2: ER membrane protein complex subunit 2; SLC7A11: Solute carrier family 7 member 11; NFE2L2: Nuclear factor erythroid 2-related factor 2; MT1G: Metallothionein 1G; HSPB1: Heat shock protein family B member 1; GPX4: Glutathione peroxidase 4; FANCD2: Fanconi anemia complementation group D2; CISID1: CDGSH iron sulfur domain 1; FDFT1: Farnesyl-diphosphate farnesyltransferase 1; SLC1A5: Solute carrier family 1 member 5; SAT1: Spermidine/spermine N1-acetyltransferase 1; TFRC: Transferrin receptor; RPL8: Ribosomal protein L8; NCDA4: NADPH cytosolic disulfide reductase 4; LPCAT3: Lysophosphatidylcholine acyltransferase 3; GLS2: Glutaminase 2; DPP4: Dipeptidyl peptidase 4; CS: Citrate synthase; ALOX15: Arachidonate 15-lipoxygenase; ACSL4: Acyl-CoA synthetase long-chain family member 4; ATP5MC3: ATP synthase membrane subunit c locus 3; CARS1: Cysteinyl-tRNA synthetase 1; ATL1: Atlastin GTPase 1.

## Results

### Ferroptosis-related gene expression and protein interaction network in ESCC

We obtained RNAseq data and corresponding clinical information from the TCGA database for 82 ESCC patients and 1456 individuals classified as normal (which also included normal controls from the GTEx database). The analysis revealed that both *NRF2* and *GPX4* were significantly overexpressed in ESCC. Moreover, 14 out of 22 ferroptosis-related genes showed elevated expression levels (cyclin-dependent kinase inhibitor 1A [*CDKN1A*], heat shock protein family A member 5 [*HSPA5*], solute carrier family 7 member 11 [*SLC7A11*], *NFE2L2*, metallothionein 1G [*MT1G*], *HSPB1*, *GPX4*, Fanconi anemia complementation group D2 [*FANCD2*], CDGSH iron sulfur domain 1 [*CISD1*], farnesyl-diphosphate farnesyltransferase 1 [*FDFT1*], *SLC1A5*, spermidine/spermine N1-acetyltransferase 1 [*SAT1*], transferrin receptor [*TFRC*], NADPH cytosolic disulfide reductase 4 [*NCDA4*], arachidonate 15-lipoxygenase [*ALOX15*], and *ACSL4*), while the expression levels of three genes (ER membrane protein complex subunit 2 [*EMC2*], glutaminase 2 [*GLS2*], and dipeptidyl peptidase 4 [*DPP4*]) were reduced (Figure 1A). The correlation circle plot depicting the relationship between ferroptosis genes confirmed a positive correlation between *NRF2* and *GPX4* in ESCC (Figure 1B). Additionally, we analyzed the differential expression of GCLM in ESCC and normal tissues, revealing elevated expression levels of GCLM in ESCC (Figure 1C).

### The CCK8 assay determines the optimal irradiation dose and detection time

**Figure 2.**
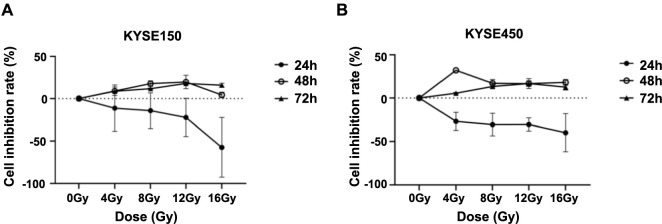
**Cell inhibition rates determined by CCK-8 assay for optimal irradiation dose and detection time.** Inhibition rates of KYSE150 (A) and KYSE450 (B) cells across various radiation doses and detection time points. CCK8: Cell Counting Kit-8.

To determine the optimal irradiation dose and detection time for the KYSE150 and KYSE450 cell lines, we used the CCK8 assay to evaluate cell inhibition rates. We set five radiation dose gradients (0Gy, 4Gy, 8Gy, 12Gy, and 16Gy) and three time points for detection (24, 48, and 72 h). Our findings indicated that the KYSE150 cell line exhibited the highest inhibition rate at 48 h with 12Gy (Figure 2A). However, statistical analysis revealed no significant difference in inhibition rates (*P* > 0.05) when compared with the 48 h, 8Gy condition. Given that a single 12Gy dose may be excessively high, we opted for 48 h, 8Gy as the standard for subsequent experiments. For the KYSE450 cell line, the most substantial inhibition rate was observed at 48 h with a 4Gy dose (Figure 2B).

### Variation in cytostatic rate after the addition of Fer-1 and TBHQ

**Figure 3.**
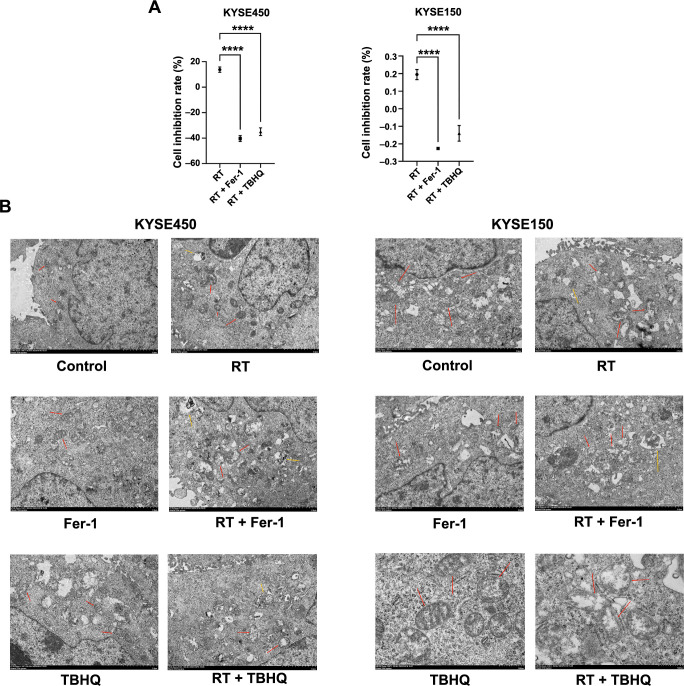
**Impact of Fer-1 and TBHQ treatment on the cytostatic rate of KYSE450 and KYSE150 cell lines.** (A) Comparison of cell inhibition rates in KYSE450 and KYSE150 cell lines subjected to three distinct treatment modalities; (B) Transmission electron microscopy images depicting morphological alterations in KYSE450 and KYSE150 cells following 12 h of radiotherapy under various treatment regimens. Red arrows indicate mitochondria, and yellow arrows highlight autophagosomes. ****P < 0.0001. Fer-1: Ferrostatin-1; TBHQ: Tert-butylhydroquinone; RT: Radiotherapy.

To demonstrate that radiotherapy can induce ferroptosis in ESCC cells, we pretreated the cells using Fer-1. CCK8 assay results showed a 54% and 42% reduction in cell inhibition in the Fer-1-treated KYSE150 and KYSE450 cell lines, respectively, compared to the radiotherapy group (Figure 3A). Additionally, transmission electron microscopy images showed characteristic morphological changes in KYSE450 and KYSE150 cells pretreated with three different measures after 12 h of radiotherapy. These changes included mitochondrial atrophy, increased membrane density, and rupture of the outer mitochondrial membrane, whereas the mitochondrial morphology of cells without radiotherapy remained essentially normal. Notably, autophagosomes with autophagic characteristics were also observed after radiotherapy (Figure 3B). Furthermore, we pretreated the cells with TBHQ for 24 h. CCK8 assay results demonstrated that the cell inhibition rates of KYSE150 and KYSE450 cells pretreated with TBHQ decreased by 48% and 33%, respectively, compared to the radiotherapy group. These alterations in cell inhibition rates suggest that radiotherapy can indeed induce ferroptosis in esophageal cancer cells, as evidenced by the reduction in cell inhibition rates with the addition of Fer-1. Moreover, this effect can be counteracted by the NRF2 agonist TBHQ.

### Detection of markers related to ferroptosis

**Figure 4.**
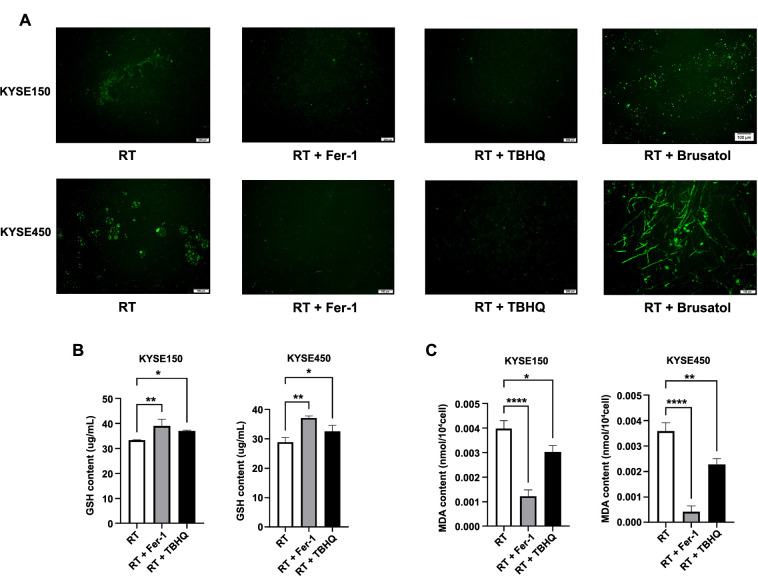
**Assessment of markers related to ferroptosis.** (A) Fluorescence imaging of ROS levels in KYSE150 and KYSE450 cell lines following four different treatments modalities; (B) Quantification of GSH expression levels in KYSE150 and KYSE450 cell lines across three different treatment modalities; (C) Quantification of MDA expression levels in KYSE150 and KYSE450 cell lines across three different treatment modalities. *P < 0.05; **P < 0.01; ****P < 0.0001. ROS: Reactive oxygen species; GSH: Glutathione; MDA: Malondialdehyde; RT: Radiotherapy; Fer-1: Ferrostatin-1; TBHQ: Tert-butylhydroquinone.

We conducted tests for several common markers of ferroptosis to further verify whether the addition of Fer-1 or TBHQ had an effect on ferroptosis. The results of the test for ROS are depicted in Figure 4A. GSH, which reflects intracellular levels of oxidative stress, was assessed. The results indicated that in the KYSE450 cell line treated with radiotherapy alone, the GSH content was the lowest. However, the GSH content significantly increased when pretreated with Fer-1 and TBHQ for 24 h, respectively (refer to Figure 4B). The trends observed in the KYSE150 cell lines were consistent with those observed in the KYSE450 cell lines. MDA, a product of membrane lipid peroxidation, was positively correlated with ferroptosis. The analysis of the results revealed that in the KYSE450 cell lines, the MDA content was highest in the radiotherapy group alone. Conversely, when Fer-1 and TBHQ were added, the degree of intracellular lipid peroxidation was inhibited, resulting in a significant decrease in MDA content (refer to Figure 4C). The trends observed in the KYSE150 cell lines mirrored those observed in the KYSE450 cell lines.

### NRF2-GCLM-GSH-GPX4 signal axis

**Figure 5.**
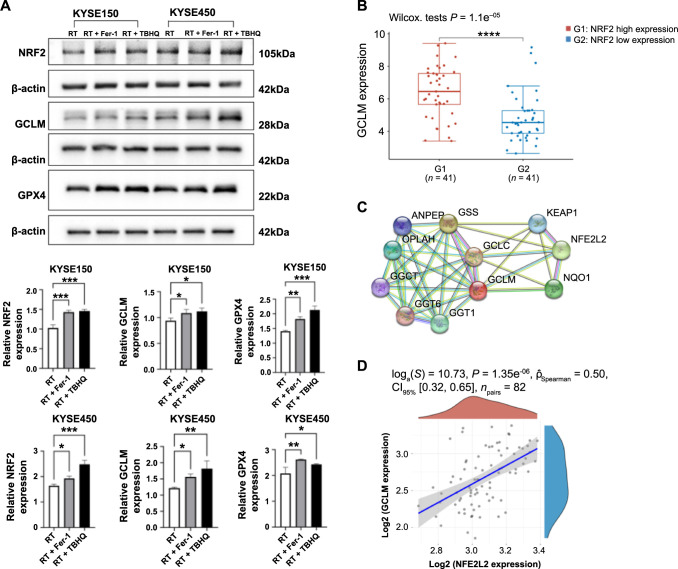
**Ferroptosis-related protein expression and interactions in ESCC.** (A) Western blot analysis results displaying the expression levels of NRF2, GCLM, and GPX4 in KYSE150 and KYSE450 cell lines, following three different treatment modalities; (B) Box plot representing NRF2 and GCLM expression levels in ESCC; (C) A protein–protein interaction network diagram illustrating the associations between NRF2 and GCLM; (D) A scatter plot detailing the correlation analysis between NRF2 and GCLM expression in ESCC. *P < 0.05; **P < 0.01; ***P < 0.001; ****P < 0.0001. ESCC: Esophageal squamous cell carcinoma; NRF2: Nuclear factor erythroid 2-related factor 2; GCLM: Glutamate-cysteine ligase modifier subunit; GPX4: Glutathione peroxidase 4; RT: Radiotherapy; Fer-1: Ferrostatin-1; TBHQ: Tert-butylhydroquinone; ANPEP: Alanyl aminopeptidase; OPLAH: 5-Oxoprolinase; GGCT: Gamma-glutamylcyclotransferase; GGT: Gamma-glutamyltransferase; GCLC: Glutamate-cysteine ligase catalytic subunit; GSS: Glutathione synthetase; Keap1: Kelch-like ECH-associated protein 1; NFE2L2: Nuclear factor erythroid 2-related factor 2; NQO1: NAD(P)H qinone dehydrogenase 1; CI: Confidence interval.

**Figure 6.**
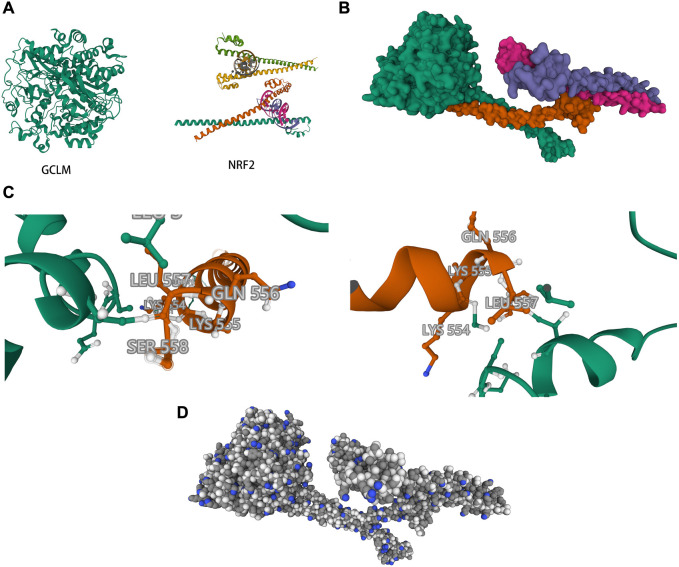
**Structural analysis of NRF2 and GCLM interactions.** (A) 3D structure of NRF2 and GCLM as found in the PDB database; (B) The refined optimal model after ZDOCK simulation, showcasing the potential docking conformation; (C) The specific binding sites where NRF2 may interact with GCLM; (D) An atomistic detail of the complex formed when NRF2 binds to GCLM. NRF2: Nuclear factor erythroid 2-related factor 2; GCLM: Glutamate-cysteine ligase modifier subunit; PDB: Protein data bank; ZDOCK: Z-domain docking; LEU: Leucine; GLN: Glutamine; LYS: Lysine; SER: Serine.

To verify the relationship between NRF2 and GCLM, we conducted further in vitro experiments. Western blot analysis revealed a significant increase in the expression of ferroptosis-related proteins in the two esophageal cancer cell lines compared to the radiotherapy group. Moreover, the expression of GCLM was positively correlated with the increase in NRF2 expression in each group. Based on these results and previous experiments, we concluded that overactivated NRF2 may affect the sensitivity of esophageal cancer cells to radiotherapy by regulating the expression of GPX4 through GCLM (Figure 5A). To further test our hypothesis, we retrieved ESCC data from the TCGA database. The results demonstrated that GCLM expression was also elevated in patients within the NRF2 high-expression group (refer to Figure 5B). Protein–protein interaction network diagrams and gene correlation analysis further supported our findings (Figure 5C and 5D). Subsequently, we downloaded the 3D structures of NRF2 and GCLM from the PDB database (Figure 6A) and conducted molecular docking simulations using ZDOCK (Figure 6B), followed by scoring. Visual analysis of ZDOCK’s optimal model score of 1151.5 revealed that NRF2 can directly form covalent bonds with residues, such as leucine (LEU) 557, lysine (LYS) 554, threonine (THR) 76, and methionine (MET) 80 of GCLM (Figure 6C), thereby achieving stable binding of the two. Figure 6D displays some details at the atomic level, including the cytoskeleton and the protein spatial structure, when NRF2 binds to GCLM.

### NRF2-mediated immune infiltration pattern in ESCC

**Figure 7.**
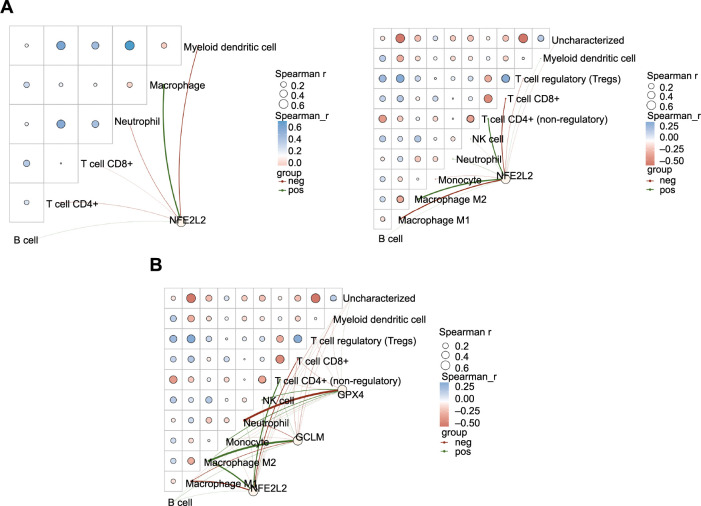
**NRF2-mediated immune infiltration pattern in ESCC.** (A) Analysis through TIMER indicating a positive correlation between NRF2 expression and macrophage infiltration degree. quanTIseq analysis revealed a significant correlation between NRF2 expression and the presence of M2 macrophages. (B) quanTIseq analysis showcasing that the expressions of NRF2, GCLM, and GPX4 show a significant positive correlation with the infiltration of M2 macrophages. NRF2: Nuclear factor erythroid 2-related factor 2; ESCC: Esophageal squamous cell carcinoma; TIMER: Tumor Immune Estimation Resource; GCLM: Glutamate-cysteine ligase modifier subunit; GPX4: Glutathione peroxidase 4; CD: Cluster of differentiation; Tregs: T cell regulatory; NFE2L2: Nuclear factor erythroid 2-related factor 2; neg: Negative; pos: Positive.

To investigate the crosstalk between NRF2 and the immune microenvironment, we analyzed the correlation between gene expression and immune scores, as well as between different components of the immune system. Utilizing the Tumor Immune Estimation Resource (TIMER), we found that NRF2 expression was positively correlated with macrophage infiltration. Furthermore, we conducted a compositional analysis using quanTIseq, which revealed a significant correlation between NRF2 expression and macrophage M2 infiltration (Figure 7A). Interestingly, quanTIseq analysis also demonstrated a positive correlation between the expression of GCLM and GPX4 and the infiltration of macrophage M2 (Figure 7B).

### Effect of NRF2-mediated massive macrophage M2 infiltration on radiotherapy resistance and ferroptosis

**Figure 8.**
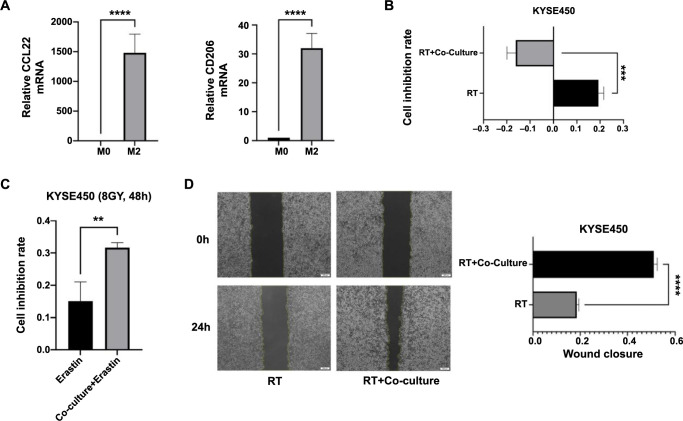
**Effects of NRF2-mediated massive macrophage M2 infiltration on radiotherapy resistance and ferroptosis.** (A) Quantitative RT-PCR analysis verifying the differentiation of THP-1 cells into M2 macrophages; (B) Graph representing the cell inhibition rates in both the co-culture and control groups after 48 h of radiotherapy; (C) Graph representing the cell inhibition rates in both the co-culture and control groups, following a 24-h pretreatment with erastin and subsequent 48-h post-radiotherapy; (D) Scratch assay images depicting the migration of cells in the co-culture and control groups at 0 h and 24 h after radiotherapy, with quantification displayed below. **P < 0.01; ***P < 0.001; ****P < 0.0001. NRF-2: Nuclear factor erythroid 2-related factor 2; RT-PCR: Reverse transcription polymerase chain reaction; CCL22: Chemokine (C–C motif) ligand 22; mRNA: Messenger RNA; CD: Cluster of differentiation; RT: Radiotherapy.

**Figure 9.**
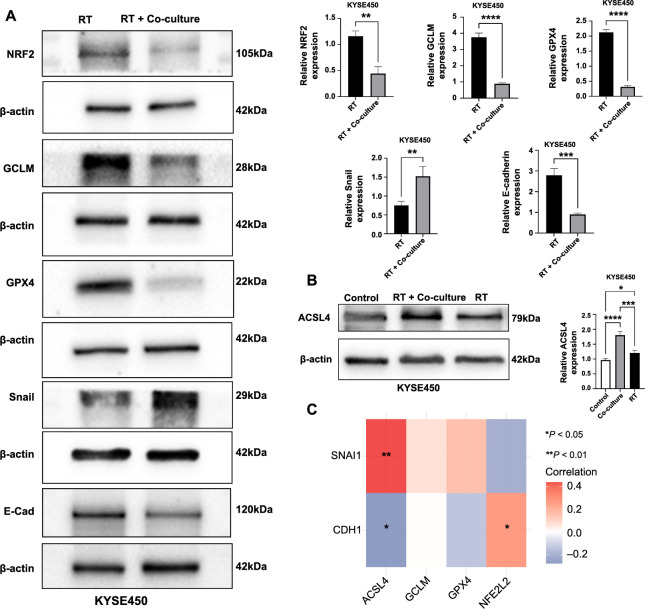
**Effects of NRF2-mediated massive macrophage M2 infiltration on radiotherapy resistance and ferroptosis.** (A) Western blot analysis comparing the expression levels of NRF2, GCLM, GPX4, Snail, and E-cadherin between RT group and the RT + co-culture group; (B) Western blot displaying the expression levels of ACSL4 in the control group, RT + co-culture group, and RT group; (C) A heatmap representing the correlation analysis between ferroptosis-related genes and EMT-related genes. *P < 0.05; **P < 0.01; ***P < 0.001; ****P < 0.0001. NRF2: Nuclear factor erythroid 2-related factor 2; GCLM: Glutamate-cysteine ligase modifier subunit; GPX4: Glutathione peroxidase 4; Snail: Snail family transcriptional repressor 1; E-cadherin: Epithelial cadherin; RT: Radiotherapy; ACSL4: Acyl-CoA synthetase long-chain family member 4; EMT: Epithelial-mesenchymal transition; E-Cad: E-cadherin; SNAI1: Snail family transcriptional repressor 1; CDH1: E-cadherin.

To explore the crosstalk between macrophages M2 and ferroptosis, we induced THP-1 cells to differentiate into macrophages M2 and co-cultured them with KYSE450 cell lines (Figure 8A). The results revealed a significant reduction in the cell inhibition rate in the co-culture group (Figure 8B). Moreover, the sensitivity to erastin, an inducer of ferroptosis, was significantly increased under the condition of radiotherapy (Figure 8C). Additionally, the scratch test results demonstrated a significantly higher cell migration rate in the co-culture group compared to the radiotherapy group (Figure 8D). Western blot analysis showed that after radiotherapy, the expression of ferroptosis-related proteins in ESCC cells in the co-culture group significantly decreased, while the expression of epithelial–mesenchymal transition (EMT)-related proteins such as E-cadherin decreased, indicating the occurrence of EMT and increased sensitivity to ferroptosis (Figure 9A). Conversely, the expression of Snail increased. Furthermore, we investigated the expression levels of the ferroptosis-related protein ACSL4, which is activated by the Hippo pathway during EMT. The results demonstrated that both treatment groups exhibited significantly increased expression compared to the control group, with the highest expression observed in the radiotherapy combined with co-culture group (Figure 9B). This suggests a potential mechanism promoting the sensitivity of esophageal cancer cells to ferroptosis inducers and radiotherapy when undergoing EMT. Additionally, we analyzed the correlation between ferroptosis-related and EMT-related proteins. NRF2 was found to be positively correlated with E-cadherin, while ACSL4 was positively correlated with Snail and negatively correlated with E-cadherin (Figure 9C). These findings align with our Western blot analysis.

### Screening of molecular targets and immune checkpoint inhibitor responses

**Figure 10.**
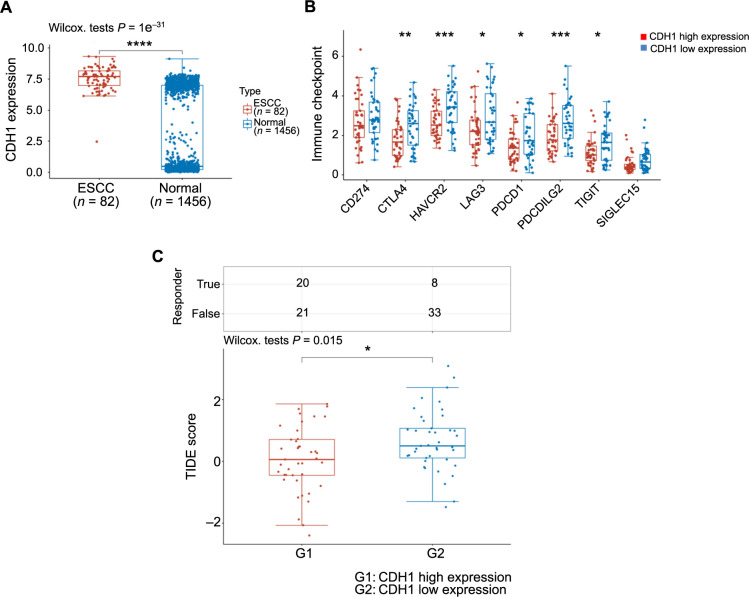
**Evaluating molecular targets and immune checkpoint inhibitor responses.** (A) Comparative expression patterns of E-cadherin in ESCC vs normal tissue samples, highlighting differential expression; (B) Analysis of immune checkpoint expression levels of E-cadherin in ESCC; (C) Predictive modeling of immunotherapy response based on E-cadherin expression in ESCC. *P < 0.05; **P < 0.01; ***P < 0.001; ****P < 0.0001. E-cadherin: Epithelial cadherin; ESCC: Esophageal squamous cell carcinoma; CDH1: E-cadherin; CD: Cluster of differentiation; CTLA4: Cytotoxic T-lymphocyte-associated protein; HAVCR2: Hepatitis A virus cellular receptor 2; LAG3: Lymphocyte activation gene 3; PDCD1: Programmed cell death protein 1; PDCD1LG2: Programmed cell death protein 1 ligand 2; TIGIT: T cell immunoreceptor with immunoglobulin and ITIM domains; SIGLEC15: Sialic acid-binding immunoglobulin-type lectin 15; TIDE: Tumor Immune Dysfunction and Exclusion.

As previously mentioned, we explored the relationship between E-cadherin and NRF2 and sought to understand the expression pattern of E-cadherin in ESCC. Our findings revealed high expression of E-cadherin in ESCC (Figure 10A). Subsequently, we conducted an immune checkpoint analysis to examine the expression of genes associated with immune checkpoints. The results indicated that the E-cadherin high-expression group exhibited lower expression of immune checkpoint-related genes (Figure 10B). Finally, we assessed the response to ICB. The results demonstrated that the TIDE score was lower in the E-cadherin high-expression group, indicating better efficacy of ICB in this group. Additionally, patients in the E-cadherin high-expression group exhibited longer survival after ICB treatment and were more likely to benefit from immunotherapy (Figure 10C).

## Discussion

As a conventional treatment for ESCC, radiotherapy primarily relies on TNM staging and employs different irradiation fields, doses, or combination chemotherapy to enhance its efficacy [21, 22]. However, the majority of ESCC patients fail to achieve long-term clinical benefits from standard radiotherapy due to the development of radiotherapy resistance, often accompanied by severe side effects [9].

Ferroptosis, being a unique form of regulatory iron-dependent lipid peroxidation-driven cell death, holds far-reaching implications in antitumor therapy. While radiotherapy can induce ferroptosis in tumor cells, and targeting ferroptosis has achieved initial results in tumor suppression [23, 24], evasion of ferroptosis mediated by specific oncogenes or oncogenic signals remains a contributing factor to tumor initiation, progression, metastasis, and treatment resistance [25, 26]. Therefore, there is an urgent need to explore the mechanisms underlying radiotherapy resistance associated with ferroptosis in ESCC, aiming to restore patients’ sensitivity to radiotherapy and improve the efficacy of individualized radiotherapy approaches.

As one of the three primary mechanisms counteracting ferroptosis, NRF2 mediates protective responses depending on the cell and tissue environment. The specific inhibitory mechanism of ferroptosis in pancreatic and liver cancer has been well established [27, 28]. However, studies investigating the mechanisms of radiotherapy-related anti-ferroptosis in ESCC are lacking.

To address this gap, we initially predicted the downstream binding protein of NRF2 through the RNA-binding protein (RBP) database. The results indicated that NRF2 could bind to the 3’ untranslated region (3’ UTR) structure of GCLM through a specific binding site. Subsequently, we evaluated the inhibitory effects of different drug pretreatments on ferroptosis induced by radiotherapy in two distinct ESCC cell lines. The cell inhibition rate of the Fer-1 treatment group was significantly lower than that of the radiotherapy group, demonstrating that radiotherapy could indeed induce ferroptosis in ESCC cells. Furthermore, the cytostatic rate of the TBHQ treatment group was significantly reduced, indicating that the radiotherapy-induced ferroptosis effect could be rescued by overexpressed NRF2. Western blot analysis revealed that among the two ESCC cell lines, the expression of three ferroptosis-related proteins was lowest in the radiotherapy group, intermediate in the Fer-1-treated group, and highest in the TBHQ-treated group. Interestingly, we observed higher expression levels of GCLM and GPX4 in the TBHQ-treated group compared to the Fer-1-treated group, suggesting that NRF2 has a more direct effect on GCLM and enhances GPX4 expression. Next, we further elucidated the binding relationship between NRF2 and its downstream protein GCLM through correlation analysis and molecular docking. This exploration led to the identification of a new radiotherapy-related ferroptosis signaling axis in ESCC, namely, the NRF2-GCLM-GSH-GPX4 signaling axis. Furthermore, we identified a new molecular target, GCLM, within this axis. The interaction between NRF2 and GCLM is consistent with the findings of Hu et al. and has been thoroughly validated in the literature [10]. We propose that NRF2 mediates the mechanism of anti-ferroptosis in radiotherapy by binding to the downstream protein GCLM. This interaction enhances GCLM’s affinity for glutamate and improves its catalytic activity, thereby reducing the intracellular peroxidation level.

TAMs, recognized as the most crucial immune cells within the tumor microenvironment, have been extensively studied for their significant role in tumor progression. TAMs have been reported to contribute to various aspects of tumor development, including promoting tumor cell proliferation, fostering immunosuppression, and facilitating angiogenesis, ultimately culminating in tumor growth and metastasis [29, 30]. However, the precise mechanisms underlying how TAMs influence ferroptosis in tumors remain elusive. EMT represents a process in which epithelial cells undergo alterations, losing their epithelial-related polarity and intercellular adhesion properties. This transition is accompanied by a gradual increase in aggressiveness and the acquisition of a more motile mesenchymal phenotype. EMT is implicated in promoting metastatic dissemination in various tumor types and contributes to the development of drug resistance during clinical treatment [31–33].

In order to investigate the impact of the TIME on radiotherapy sensitivity in ESCC, we examined the immune infiltration pattern of NRF2 and observed a positive correlation between NRF2 and macrophage M2. While significant negative associations have been reported between tumor ferroptosis and macrophage M2 infiltration [19, 34, 35], few studies have explored the direct effects of macrophage M2 on ferroptosis in tumors. To further elucidate the crosstalk between macrophage M2 and radiotherapy, we co-cultured KYSE450 cell lines with macrophage M2. The results of cell proliferation assays and scratch assays revealed that ESCC cells exhibited significantly increased resistance to radiotherapy after co-culture with macrophage M2. Additionally, these cells displayed a greater tendency for metastasis. However, interestingly, they also demonstrated increased sensitivity to ferroptosis inducers during this process. The western blot results revealed an increase in Snail expression and a decrease in E-cadherin expression, indicating that co-culture promoted the development of EMT in ESCC cells. These findings are consistent with the results reported by Song et al. [36]. Additionally, the expression of ferroptosis-related proteins was downregulated, suggesting that co-culture restored the sensitivity of tumor cells to radiotherapy-induced ferroptosis.

To explore how macrophage M2 restores the sensitivity of tumor cells to radiotherapy-induced ferroptosis through EMT, we investigated the expression levels of the ferroptosis-related protein ACSL4, which is activated by the Hippo pathway during EMT. Initially, we confirmed that radiotherapy could induce ACSL4 expression. Subsequently, we observed that ACSL4 expression levels were elevated in the co-culture combined with the radiotherapy group. The upregulation of ACSL4 expression following co-culture suggests that EMT may influence the sensitivity of ESCC cells to ferroptosis by activating the yes-associated protein 1 (YAP1) and the WW domain-containing transcription regulator protein 1 (WWTR1) in the Hippo pathway [37]. These transcription factors are known regulators of the *ACSL4* gene, thus enhancing its expression. ACSL4 integrates more polyunsaturated fatty acids into the cell membrane, thereby increasing the substrate for ferroptosis and influencing the sensitivity of radiotherapy to ferroptosis. Previous studies have demonstrated that EMT can regulate ferroptosis by modulating cell density, and activation of the Hippo pathway can inhibit ferroptosis and promote tumor development [38]. Similar findings have been observed in head and neck cancer and gallbladder cancer, where EMT appears to influence selective susceptibility to ferroptosis through various mechanisms. One such mechanism involves the high baseline transcriptional levels of zinc finger E-box-binding homeobox 1 (ZEB1), which have been implicated in inducing the upregulation of peroxisome proliferator-activated receptor gamma (PPARγ), which serves as the main regulator of liver lipid metabolism [39]. Furthermore, the lysine-rich CEACAM1 co-isolated protein (LYRIC) has been identified as a positive regulator of EMT. LYRIC promotes ferroptosis by inhibiting the expression of GPX4 and solute carrier family 3 member 2 (SLC3A2) [40]. In summary, NRF2 exhibits a dual role in the context of radiotherapy resistance and ferroptosis in ESCC. On the one hand, NRF2’s unique antioxidant mechanism contributes to radiotherapy resistance, and on the other hand, NRF2 promotes EMT in ESCC cells, facilitated by the infiltration of a large number of macrophage M2, leading to metastasis, tumor progression, and reduced sensitivity to radiotherapy. However, this process also partially restores the sensitivity of ESCC cells to ferroptosis, potentially rendering metastatic cancer cells more susceptible to ferroptosis inducers. Consequently, this could enhance the efficacy of radiotherapy in killing tumor cells. Additionally, epigenetic reprogramming associated with EMT may facilitate ferroptosis in ESCC cells, presenting a novel avenue for immunotherapy targeting ferroptosis.

Subsequently, we conducted an analysis of the correlation between ferroptosis-related genes and EMT-related genes. Our findings revealed a positive correlation between E-cadherin expression and NRF2. Following this, we examined the expression levels of immune checkpoint-related genes in patients exhibiting these two distinct expression patterns. Interestingly, we observed a downregulation of six out of eight common immune checkpoints in ESCC patients with high E-cadherin expression. Furthermore, we stratified ESCC patients into high- and low-expression groups based on E-cadherin gene expression levels. Subsequently, we assessed the response to ICB therapy in these patient groups. The results showed that patients in the high-expression group exhibited lower scores, indicating a higher likelihood of benefiting from immunotherapy in that group. However, several limitations exist within our study. Firstly, the bidirectional role of ACSL4 in EMT and ferroptosis remains unclear. Secondly, our study was validated to in vitro cell experiments and lacked validation in animal models. The complex internal environment of animals may introduce variables that could potentially distort our conclusions. Moreover, our analysis of immunoinfiltration relied on bioinformatics tools, such as TIMER and quanTIseq. While supported by the existing literature, the reliance on computational methods without in vitro experiments or clinical analyses may introduce bias and compromise the accuracy of our conclusions. Moving forward, it is imperative to further elucidate the role of macrophage M2 in ESCC through extensive basic experiments. Additionally, investigating how EMT affects ferroptosis and therapeutic sensitivity warrants further exploration. Furthermore, both in vivo and in vitro experiments are needed to validate the reliability and safety of ICB therapy.

## Conclusion

Overall, our study revealed a novel signaling axis involving NRF2-mediated regulation of ferroptosis in ESCC, shedding light on its implications for radiotherapy sensitivity. Furthermore, we elucidated the mechanism by which NRF2 modulates macrophage M2 infiltration, influencing ferroptosis and radiotherapy sensitivity. These findings offer novel insights into potential targets for targeted therapy and immunotherapy in ESCC.

## Data Availability

The data supporting the findings of this study are available from the authors upon reasonable request. Additionally, the data has been uploaded to: https://www.scidb.cn/en/anonymous/ZlU3QnJx.
